# Colonic microbiota is associated with inflammation and host epigenomic alterations in inflammatory bowel disease

**DOI:** 10.1038/s41467-020-15342-5

**Published:** 2020-03-23

**Authors:** F. J. Ryan, A. M. Ahern, R. S. Fitzgerald, E. J. Laserna-Mendieta, E. M. Power, A. G. Clooney, K. W. O’Donoghue, P. J. McMurdie, S. Iwai, A. Crits-Christoph, D. Sheehan, C. Moran, B. Flemer, A. L. Zomer, A. Fanning, J. O’Callaghan, J. Walton, A. Temko, W. Stack, L. Jackson, S. A. Joyce, S. Melgar, T. Z. DeSantis, J. T. Bell, F. Shanahan, M. J. Claesson

**Affiliations:** 1 School of Microbiology, University College Cork, Cork, Ireland; 2 APC Microbiome Ireland, University College Cork, Cork, Ireland; 3 Department of Biological Sciences Cork Institute of Technology, Cork, Ireland; 4 Second Genome, South San Francisco, CA 94080 USA; 5 Department of Medicine, University College Cork, Cork, Ireland; 6 Radboud University Medical Center, Laboratory of Pediatric Infectious Diseases, Nijmegen, The Netherlands; 7 School of Food and Nutritional Sciences, University College Cork, Cork, Ireland; 8 Irish Centre for Fetal and Neonatal Translational Research, Department of Electrical and Electronic Engineering, University College Cork, Cork, Ireland; 9 Bon Secours Hospital, Cork, Ireland; 10 School of Biochemistry and Cell Biology, University College Cork, Cork, Ireland; 11 Department of Twin Research and Genetic Epidemiology, King’s College London, London, UK

**Keywords:** Data processing, Epigenomics

## Abstract

Studies of inflammatory bowel disease (IBD) have been inconclusive in relating microbiota with distribution of inflammation. We report microbiota, host transcriptomics, epigenomics and genetics from matched inflamed and non-inflamed colonic mucosa [50 Crohn’s disease (CD); 80 ulcerative colitis (UC); 31 controls]. Changes in community-wide and within-patient microbiota are linked with inflammation, but we find no evidence for a distinct microbial diagnostic signature, probably due to heterogeneous host-microbe interactions, and show only marginal microbiota associations with habitual diet. Epithelial DNA methylation improves disease classification and is associated with both inflammation and microbiota composition. Microbiota sub-groups are driven by dominant *Enterbacteriaceae* and *Bacteroides* species, representative strains of which are pro-inflammatory in vitro, are also associated with immune-related epigenetic markers. In conclusion, inflamed and non-inflamed colonic segments in both CD and UC differ in microbiota composition and epigenetic profiles.

## Abstract (web summary)

Inflammatory bowel disease (IBD) has been linked to host-microbiota interactions. Here, the authors investigate mucosa-associated microbiota using endoscopically-targeted biopsies from inflamed and non-inflamed colon in patients with Crohn’s disease and ulcerative colitis, finding associations with inflammation and host epigenomic alterations.

## Introduction

The chronic inflammatory bowel diseases (IBD), Crohn’s disease (CD), and ulcerative colitis (UC), are heterogeneous disorders with distinct and overlapping features that represent the outcome of abnormal host-microbe interactions, in genetically susceptible individuals. While the pathogenesis of IBD in experimental models highlight the role of host-microbe interactions, human studies are less clear and inconsistent, without a definitive cause-effect relationship. Small study populations and protocol variations have confounded interpretation and comparative analyses of human studies. Studies of the mucosa-associated microbiota are likely to be more informative than those of fecal microbiota in relation to host-microbe interactions. Moreover, although habitual diet is known to be an important determinant of the composition and metabolic activity of the gut microbiota, dietary analysis has been lacking from several studies of the microbiota in IBD. Furthermore, the relationship between mucosal inflammation, the microbiota and the epigenome has received little attention^1^. For these reasons, we here investigated mucosa-associated microbiota using endoscopically-targeted biopsies from paired inflamed and non-inflamed segments of the colon in patients with Crohn’s disease and ulcerative colitis. The results show that the microbiota in both forms of IBD exhibit reduced diversity and increased variability compared with the microbiota of controls, and host fewer bacteria from Clostridium cluster XIVa, *Anaerostipes hadrus* and an unclassified species of the Lachnospiraceae family. While there is substantial overlap in these features for CD and UC microbiota, they are most evident in CD. We observe community-wide and within-patient changes in microbiota composition between inflamed and non-inflamed colonic mucosa, but these are not attributed to specific taxa. As expected, host mucosal gene expression is most discriminatory of inflammation, but is followed by host DNA methylation and microbiota composition. While microbiota composition alone cannot robustly classify disease, it stratifies the subjects into sub-groups with different epigenetic profiles.

## Results

### Microbiota is associated with disease and inflammation

**Fig. 1:**
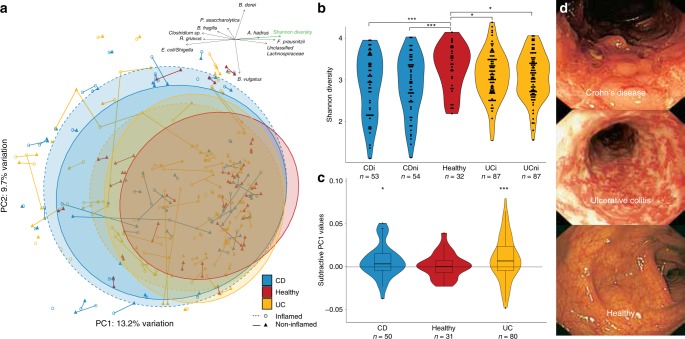
Overall microbiota composition and diversity of inflamed and non-inflamed colonic mucosa from 161 subjects (CD—blue, UC—yellow, and healthy controls—red), based on 257 RSVs that were present in ≥5% of the samples. **a** PCoA of Bray–Curtis distances with paired biopsies from each subject connected by vectors. Ellipses indicate 80% confidence regions with solid and dashed lines for non-inflamed and inflamed mucosa, respectively. The top arrows represent gradients of Shannon diversity (green) and abundances of a selection of bacterial taxa most correlated with the first two principal coordinates (Supplementary Table 2). **b** Differences in Shannon diversity were significantly lower in IBD compared with healthy mucosa (unpaired biopsies included), but not between diseases or for different inflammation status (Mann–Whitney test, two-sided; *P*-values: CDi vs H:0.006; CDni vs H: 0.008; UCi vs H: 0.0102; UCni vs H: 0.050). **c** For each pair of biopsies PC1, values from the inflamed sample were subtracted from the non-inflamed sample for each of the three conditions (reference samples randomly selected for healthy controls). Medians significantly higher than zero indicate within-patient gradient of inflammation away from non-inflamed/healthy microbiota (one-sample Wilcoxon, two-sided; *P*-values: CD = 0.08; UC = 0.009; box plot lower and upper sides show 25th and 75th percentiles, respectively. The whiskers are 1.5 of the interquartile range. *P*-values: *<0.1; **<0.05; ***<0.01). **d** Representative photographs of colons from CD, UC, and control subjects. Source: ref. ^86^.

**Fig. 2:**
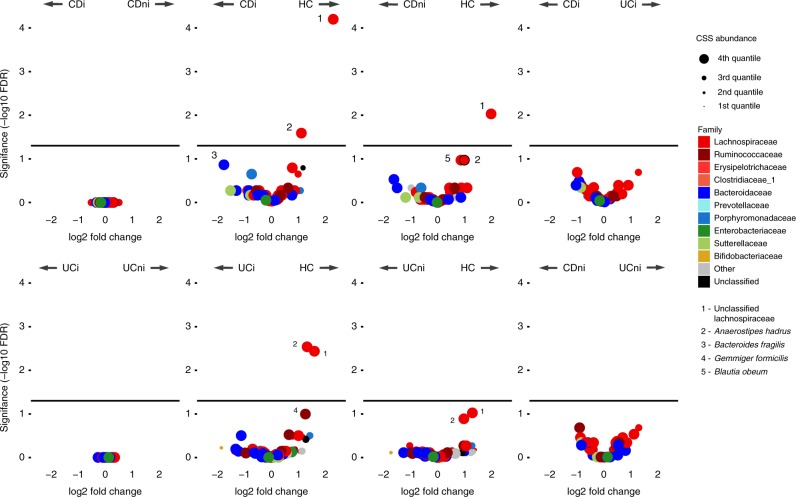
Volcano plots showing differential abundance of RSVs between sample groups. Both inflamed vs. non-inflamed tests were based on paired biopsies only. *y*-axis show adjusted *P* value (false discovery rate) and *x*-axis shows log2 fold change. Horizontal lines reflect 0.05 FDR. Points are colored by family level classification based by Mothur against the RDP database v11.4. Circle sizes are assigned based on the mean cumulative-sum scaling (CSS) and divided into quartiles with the larger circles corresponding to higher abundances. Species discussed in the text are explicitly listed; see Supplementary Table 3 for a complete list.

We studied paired biopsies from inflamed and non-inflamed mucosa of 80 adult patients with ulcerative colitis and 50 with Crohn’s disease, along with paired biopsies of 31 non-IBD (here: healthy) controls (Table 1; Supplementary Table 1), all recruited in Ireland. Microbiota composition of 346 colonic biopsies were analyzed from a total of 8,443,723 quality-filtered Illumina MiSeq reads of the amplified 16S rRNA V3-V4 gene region, with a mean of 24,466 ± 1272 (95% CI) reads per biopsy. From these amplicons, 3222 unique, error-corrected and chimera-free ribosomal sequence variants (RSVs) were generated. We carried out an unsupervised principal coordinate analysis (PCoA) of Bray–Curtis distances from the 257 RSVs that were present in ≥5% of the samples (Fig. 1a). The PCoA showed greater variation (spread) for CD compared with UC, which in turn had higher variation than healthy microbiota. While 50% of the CD and UC samples were found within the 80% confidence region of the healthy cohort, the remaining IBD samples displayed a shift away from the healthy microbiota as demonstrated by significantly lower PC1 values (*p*-value ≤0.05). An observable and significant gradient of increasing diversity in the direction of healthy samples was nearly parallel with the PC1 axis (Fig. 1a). Abundances of a number of specific bacterial taxa also correlated with the principal coordinates (Fig. 1a; Supplementary Table 2). An *Escherichia*/*Shigella*/*Klebsiella* RSV was decreasing in abundance with PC1, whereas *Faecalibacterium prausnitzii*, on the other hand, had seven RSV that were increasing along this axis, supporting previously reported lower diversity of such sub-species in IBD mucosa^2^. *Bacteroides dorei* were more common for samples with higher PC2 values, while *B. vulgatus* abundances were higher for samples with lower PC2 values. These anti-correlated within-genera differences emphasize the importance of species-level classification of 16S rRNA gene sequences. A small number of these RSVs were also found to have significantly different abundances between disease statuses. Neither disease duration, age nor gender were correlated with these PCs. Overall, we found only two RSVs that were differentially abundant with false discovery rates (FDR) lower than 5% (Fig. 2; Supplementary Table 3). An RSV from the Lachnospiraceae family [labeled 1 in Fig. 2] was less abundant in UC or CD mucosa than in healthy controls. Another RSV belonging to the Lachnospiraceae family, butyrate-producing *Anaerostipes hadrus* [labeled 2] was also less abundant in UC or CD mucosa than in healthy controls. These observations were more evident in CD than in UC, and in inflamed mucosa compared with non-inflamed. A *B. fragilis* RSV [labeled 3] was more abundant in inflamed CD mucosa than in healthy mucosa (FDR = 0.137), while *Gemmiger formicilis* [labeled 4] was less abundant in inflamed UC compared with controls (FDR = 0.101).

**Table 1:** Subject characteristics across the three cohorts.

	Crohn’s disease	Ulcerative colitis	Healthy controls
Number of biopsy pairs	50	80	31
Total number of biopsies^a^	108	174	63
Mean age (range)	43.1 (21–79) yrs	47.6 (20–76) yrs	56.9 (29–74) yrs
Gender (M/F)	28/26	46/41	18/14
Mean time since diagnosis (range)	11.4 (0–40) yrs	10.4 (0–30) yrs	N/A
% relapsing patients within 24 months of biopsy	32.7%	37.9%	N/A
% smokers (ex-smokers)	18.5% (1.9%)	4.9% (2.5%)	3.4 (3.4%)
*No. of patients on medication*			
5-aminosalicylic acid	8	59	N/A
Corticosteroids	5	22	N/A
Anti-TNF	7	8	N/A
Mercaptopurine	14	11	N/A
Antibiotics^b^	2	0	0

^a^Includes additional unpaired biopsies where matching biopsies had been excluded for technical reasons.

^b^Antibiotics taken at the time of sampling.

Even though patients with CD and UC had lower microbiota diversities than healthy individuals, inflammatory status per se did not appear to affect diversity levels (Fig. 1b). For most patients, biopsies from the same colon had similar microbiota composition regardless of inflammatory status, as illustrated by paired samples being only a short distance away (Fig. 1a). However, the overall compositional profiles for inflamed biopsies were directed away from their non-inflamed counterparts, along a gradient that deviated from healthy microbiota. To examine whether these overall shifts were also present within subjects, we subtracted non-inflamed from inflamed PC1 values for each subject, as the observed inflammation gradient was present for both CD and UC samples along this principal coordinate. Figure 1c shows these subtractive values being significantly higher than zero for both IBD cohorts, while not for the healthy cohort. No such change was observed for the subsequent 20 principle coordinates. In spite of these community-wide and subject-specific compositional differences, no individual taxa were found to be significantly abundant in inflamed relative to non-inflamed mucosa in either disease (Fig. 2).

Similarly to taxonomic composition, we did not find any differences in terms of inferred encoded function^3^ between inflamed and non-inflamed microbiota. We did, however, find 28 and 30 differentially abundant KEGG Orthologs for CD and UC, respectively, compared with controls (Supplementary Table 4). Among these, NADH dehydrogenase genes (oxidative phosphorylation pathway) are more common in UC and CD, as inflamed environments produce endogenous molecules, including oxygen, to be used as terminal electron acceptors by facultative or obligate aerobic bacteria like Enterobacteriaceae^4,5^.

### Microbiota clusters are driven by dominant species

**Fig. 3:**
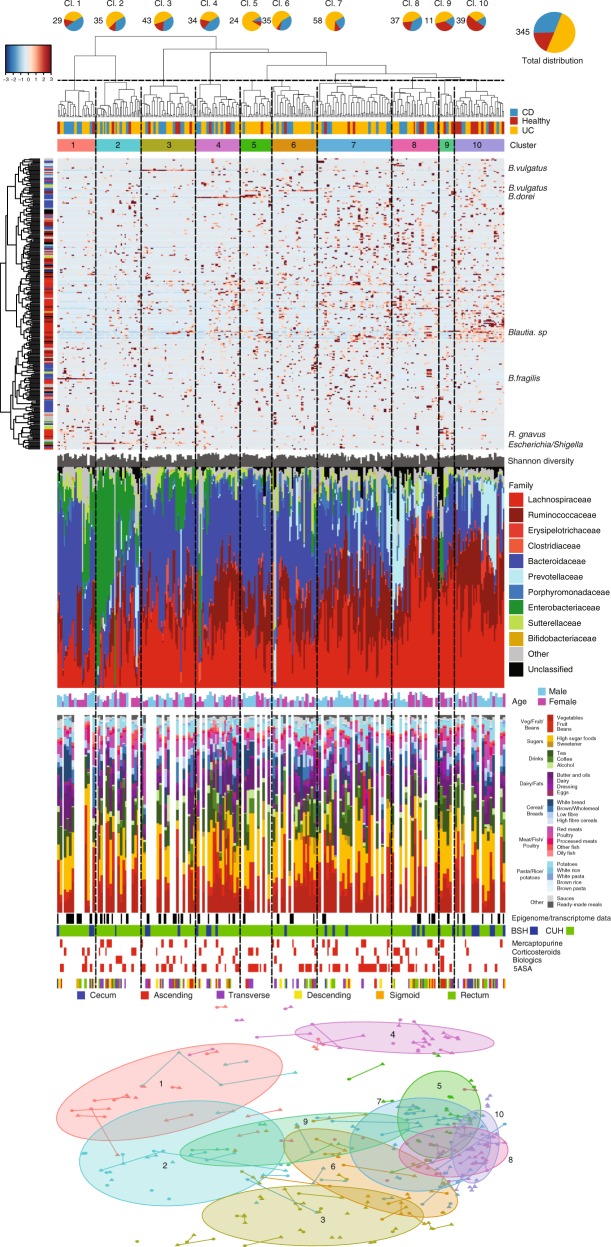
Sample clustering, diversity and relative abundance of mucosal microbiota from 346 biopsies (CD, UC and healthy), based on 257 ribosomal sequence variants (RSVs) that were present in ≥5% of the samples. From the top: pie charts with total numbers and proportions of the three subject cohorts for each cluster; hierarchical Ward linkage clustering based on Bray–Curtis distances; cohort belongingness. Ten individually colored clusters obtained through the DynamicTreeCut algorithm; heatmap with RSV abundance values to the right of vertical clustering of RSVs using Ward linkage based on Spearman correlation coefficients (heatmap shows *z*-scores, i.e., number of standard deviations from the mean value of each row); Shannon diversity for each sample; and bar plot of relative abundances at taxonomic family levels with red families belonging to the Firmicutes phylum, blue Bacteroidetes, green Proteobacteria, and yellow Actinobacteria; age and gender for each sample, major food categories, hospital, medication, and biopsy location; clusters mapped back onto the Bray–Curtis PCoA from Fig. 1a. The right-most margin shows species classifications for RSVs consistently abundant for certain clusters.

Hierarchical clustering and dynamic tree cutting of microbiota compositions based on 257 RSVs resulted in 10 sub-clusters that by visual inspection also corresponded well to relative family abundances (Fig. 3). Each of these clusters had different proportions of subject cohorts, and for the majority (≥95%) of the paired samples, were directly adjacent and within the same cluster. No cluster consisted entirely of one subject cohort, with healthy individuals present in all, as expected from the dispersed and heterogenous cohort distribution in Fig. 1a. Adjacent clusters 9 and 10 had disproportionally more healthy subjects than other clusters, whereas clusters 1, 2, and 5–7 had disproportionally more subjects with CD or UC. Notwithstanding the significant inter-individual variability of family abundances, there was an overall increase in Firmicutes:Bacteroidetes ratio in the direction of cluster 1–10, which correlated with both Shannon diversity (*R*^2^ = 0.37; *p*-value = 3.2e−12) and increasing PC 1 values (*R*^2^ = 0.44; *p*-value = 2.2e−16) with the exception of clusters 6 and 9. Particular taxonomic families dominated a number of outlier samples, some of which associated to treatment. The two patients with CD who were on antibiotics (Augmentin/Metronidazole and Azithromycin) at the time of sampling belonged to cluster 2 (left and middle), with unusually high levels of Enterobacteriaceae (89.8% and 32.1%, respectively). Of the 15 patients on biologics (anti-TNFs: Adalimumab and Infliximab), one CD and one UC patient in clusters 3 and 6 had very low-Firmicutes abundance, 7.1% and 2.5%, respectively. The low-Firmicutes (11.6%) UC patient in cluster 4 was on corticosteroids and diarrhea medication. No other subjects on similar medications showed outlier behavior in terms of relative taxa abundance.

Strikingly, seven RSVs were consistently in high abundances in five clusters (Fig. 3 and Supplementary Fig. 1): *B. fragilis* was enriched in cluster 1, *B. vulgatus* in cluster 3 and *B. dorei* in cluster 4. Their correlations with PC1 and PC2 (Fig. 1a) also explain their cluster separation. Similarly, taxa known to be associated with IBD, *Escherichia*/*Shigella*/*Klebsiella*^6^ and *Ruminococcus gnavus*^7^, were highly abundant in all cluster 2 and 9 samples, respectively, which all had relatively low PC1 values. Statistical testing indicated that no experimental batch effects were causative of these taxa enrichments (Supplementary Table 14). To investigate the effect of these cluster-dominant species on the overall microbiota composition we re-drew the PCoAs in Figs. 1 and 3 after the seven RSVs were removed one-by-one (Supplementary Fig. 2). We only observed notable changes to the five confidence regions in Fig. 1a when the *Escherichia*/*Shigella*/*Klebsiella* RSV was removed, which in effect canceled out the inflammation-associated gradient for UC. The effect of the sequential removal of these RSVs was, however, larger for some of the 10 clusters mapped on the PCoA, clearly emphasizing how single RSVs can drive microbiota-based subject stratification. Here, removing *B. fragilis* (enriched in cluster 1) caused a bigger overlap with clusters 1 and 2. Removal of the *Escherichia*/*Shigella*/*Klebsiella* RSV (cluster 2) significantly reduced the separation of that cluster with most other clusters. *Bacteroides* RSVs *B. vulgatus* (cluster 3) and *B. dorei* (cluster 4) were already anti-correlated with the PC2 axis (Fig. 1a), and their subsequent removal either reversed, or drastically changed, the clusters’ positions along that axis. We further investigated whether the four more pronounced clusters 1–4 were supported by host-related molecular data.

### Microbiota is associated with host epigenome

**Fig. 4:**
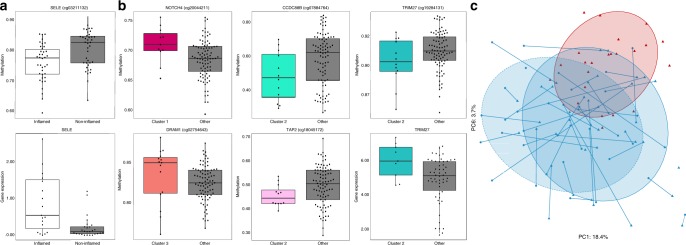
Differences in host DNA methylation and gene expression across subject groups with regards to inflammation and microbiota clustering. Significant differential host DNA methylation [beta values; significance determined using mixed linear models from lme4 library, adjusted with FDR] with corresponding gene expression [log2(fragments per kilobase of transcript per million mapped reads)] of examples of immune-related genes in **a** inflamed/non-inflamed CD tissue, **b** microbiota clusters 1–3 (expression, using *stattest* from the ballgown library, was significant before adjustment for multiple testing with FDR, and for a subset of 71 matching samples; extreme outliers removed to improve clarity; gene body methylation in NOTCH4, DRAM1, and TRIM27 (**b**), promoter methylation in SELE (**a**) and CCDC88B (**b**)) (box plot lower and upper sides show 25th and 75th percentiles, respectively. The whiskers are 1.5 of the interquartile range). **c** Epigenome principal component analysis outlining the inflammation- (PC1) and disease- (PC6) associated epigenetic trends.

To explore underlying host-microbe interactions, we assessed host epigenomics (genome-wide DNA methylation) analysis on a subset of 100 biopsies from controls (23 unpaired samples) and patients with CD (77 samples, whereof 72 paired) with matching microbiota and host epigenome. Of these, we also had matching host transcriptome data from 71 samples. Overall, the inflammation-associated epigenomes had lower PC1 values compared with both the non-inflamed and control samples (ANOVA *p*-value < 3.27e−04; Supplementary Fig. 4), indicating a stronger inflammation-related gradient compared with the microbiota (Fig. 1a). There was also a strong correlation (*R*^2^ = 0.87; ANOVA *p*-value < 4.4e−16; Supplementary Fig. 4) between the transcriptome and epigenome PC1 values for the same samples. This observation was supported by 221 genes differentially expressed between inflamed and non-inflamed tissue and associated with hyper/hypo-methylated CpG sites within or immediately adjacent to the gene (*p*-values < 0.05 in both datasets; Supplementary Table 5). Of these, the endothelial leukocyte adhesion gene Selectin E, was overexpressed and exhibited promoter hypo-methylation specifically in inflamed tissue, and has previously been implicated in inflammatory responses^8^ (Fig. 4a). While the first epigenomic principal component captured inflammation well (18.4% of total variation), the 6th epigenomics principal component (3.7% of total variation) showed significant overall methylation differences between CD (irrespective of inflammation) and healthy controls (*p*-value < 2.7e−08; Fig. 4c). There were, however, no significantly methylated sites corresponding to this observation after adjustment for multiple testing.

The biopsies were randomly selected prior to microbiota clustering (Fig. 3), and since approximately half of them were from microbiota clusters 1–4, methylation in these samples were sequentially compared with all the other clusters combined, due to fewer samples with methylation and gene expression data in clusters 5–10. To reduce the effect of different cell type compositions associated with inflamed tissue, we analyzed each set of significant epigenetic signals reported for the clusters, incorporating the first 10 PCs as covariates in the linear model which together explain 52% of methylation capturing a large extent of cell heterogeneity (see Methods). Of the 734 sites (523 of which were associated with annotated (498 unique) genes, whereof the top 12 in Supplementary Fig. 5) that were significant after PC correction (*p*-value < 0.05; Supplementary Table 6), we observed 33 and 19 significantly hyper-methylated or hypo-methylated sites in cluster 1, respectively, including hyper-methylated signals in the gene body of NOTCH4 within which mutations have been associated with IBD^9^ (Supplementary Table 6, Fig. 4b). Cluster 2 exhibited a much larger number of differentially methylated CpG sites (131 hyper- and 475 hypo-methylated), including hypo-methylation in CCDC88B (recently correlated with risk of CD^10^) and TAP2 (involved in genetic heterogeneity of CD^11^). Possible interaction of methylation and gene expression was exemplified with TRIM27, which was simultaneously overexpressed (prior to FDR-adjustment) and hypo-methylated (Supplementary Table 6, Fig. 4b). This gene negatively regulates the NOD2 gene, which if mutated can promote IBD^12^, and has been shown to promote tumor growth in DSS-induced mice if overexpressed^13^. Of the 23 hyper- and 18 hypo-methylated sites in cluster 3, significant hyper-methylation was observed in the gene body of DRAM1, also involved in NOD2 signaling^12^. Taken together, these findings identify a large number of CpG sites that are differentially methylated across the microbiota-defined clusters, whereof many in proximity to genes known to play a role in IBD.

Based on the mucosal DNA of all subjects, we investigated whether the 264 reported gene loci associated with IBD^14–28^ in the present study. While the sample size was insufficient for a Genome-Wide Association Study, a principal component analysis on nominal categorical data of the known IBD loci indicated a significant overlap of the three cohorts, albeit with a slight shift along the second component for UC (Supplementary Fig. 3a).

### Inflammation-related host expression and in vitro validation

We further analyzed the host mucosal transcriptome using a polyadenylated capture and RNA-Seq approach resulting in 13,237,135 ± 1,578,996 (95% CI) mRNA reads per biopsy. Like the epigenome, the transcriptome displayed clearly noticeable differences between inflamed and non-inflamed mucosa for both diseases, with a shift away from healthy transcriptomes along PC1 (Supplementary Fig. 4). These overall differences were translated to 2171 (out of 17,461 in total) transcripts with significantly different expression levels between inflammation statuses for the CD patients (Supplementary Table 7) and 4154 for the UC patients (Supplementary Table 8), with an overlap of 1146 transcripts. An enrichment analysis of Gene Ontology biological processes showed that genes overexpressed in inflamed UC and CD relative to non-inflamed mucosa were consistent with positive regulation of a general innate and adaptive immune response toward microbes, mediated by increased cytokine production and a corresponding inflammatory response. (Supplementary Tables 9–10). In particular, 136 (UC) and 246 (CD) transcripts known to be involved in positive regulation of cytokine production were overexpressed in inflamed mucosa, including pathways for the production of IL-1, IL-2, IL-4, IL-6, IL-8/CXCL8, IL-10, IFNγ, and TNF, which were also upregulated in other IBD studies^29,30^. Furthermore, 118 (UC) and 118 (CD) transcripts were enriched for the defense response to other organisms, 51 (UC) and 108 (CD) transcripts were involved in the response to lipopolysaccharide, and 113 (UC) and 46 (CD) transcripts for the response to molecules of bacterial origin, all indicating a pathogenesis consistent with excessive anti-microbial immunological activity. Several pathways indicating an active immune response, including positive regulation of neutrophil migration, positive regulation of leukocyte migration, and T cell migration, were also upregulated in UC and CD inflamed mucosa. Over 90% of the GO biological processes enriched in inflamed CD mucosa were also enriched in inflamed UC mucosa. Biological processes found enriched exclusively in inflamed CD transcripts were mostly involved in positive regulation of blood circulation and the vascular system (Supplementary Tables 11). The 53 gene transcripts significantly underexpressed in inflamed compared with non-inflamed UC mucosa were primarily involved with cellular respiration, mitochondrial electron transport chain activity, and ATP synthesis (Supplementary Tables 12). A recent study also indicated suppressed mitochondrial gene expressions in active UC in a pediatric cohort^31^. Of the 28 underexpressed transcripts involved in ATP synthesis coupled electron transport are genes encoding subunits of Cytochrome c oxidase, which suggests a role for mitochondrial dysfunction in the pathogenesis of active UC inflammation (Supplementary Tables 12). Thus, epithelial cells that have comparatively high mitochondrial content may significantly contribute to transcriptomic fold-changes in non-inflamed material, consistent with their relative dilution by other cells and/or loss in IBD-related inflammation. No evidence for mitochondrial dysfunction was found in the CD differential gene set.

To corroborate epigenome-host-microbiota findings, intestinal epithelial cells (IECs) were co-cultured with type strains representing dominant bacteria species from the identified microbiota clusters with differential epigenome profiles (cluster 1: *B. fragilis* ATCC25285; cluster 2: *E. coli* AIEC-HM605; cluster 3: *B. vulgatus* ATCC8482). The well-known gastrointestinal pathogen *S. typhimurium* (ATCC) and the non-inflammatory *Lactobacillus rhamnosus* (LGG) was included as positive and negative controls, respectively. We observed a significantly higher secretion of IL-8/CXCL8 (Supplementary Fig. 6), a neutrophil chemokine commonly correlated with active disease in IBD^32^ and higher CCL20/MIP3A (Supplementary Fig 6), a chemokine strongly chemotactic for lymphocytes and enhanced in active IBD^33^, for *B. vulgatus*, AIEC-HM605 and *S. typhimurium* when compared with untreated and LGG-treated cells.

### Multi-omics integration and classification

The impact of diet on the microbiota composition was assessed using a semi-quantitative food frequency questionnaire^34,35^. No major correlations were noted, as shown in the Supplementary Data. Similarly, we found no medication to be significantly more common across any of the 10 microbiota clusters (Supplementary Fig. 3).

We finally tested whether various data type combinations could improve classification of disease and inflammation status, using the Machine Learning technique Extreme Gradient Boosting^36^. Microbiota combined with diet and host genotype were better at classifying between CD, UC, and healthy status (AUC = 0.75; *p*-value ≤ 0.001) than any other combination of these data types (Supplementary Fig. 8 and Supplementary Table 13). Interestingly, adding epigenomes improved AUCs even further, up to 0.87 (*p*-value ≤ 0.001) together with microbiota only. The highest classification weight is carried by CpG sites of the PTPRO/TRIM31 genes and *A. hadrus* as indicated above. Similarly, epigenome data adds classification power to distinguishing CD inflamed from non-inflamed tissue (AUC range: 0.72–0.86), and more so than transcriptome data (AUC range: 0.63–0.75). In terms of classifying inflammation in UC, adding host transcriptome data for only 12 UC samples allows for a markedly increased AUC to 0.83 (*p*-value ≤ 0.01) over “microbiota + genotype + diet”.

## Discussion

The results confirm changes in the microbiota of patients with IBD in terms of reduced diversity and increased variability of colonic microbiota^37–39^, particularly in Crohn’s disease and to a lesser degree in ulcerative colitis. However, differences between these two forms of IBD and between inflamed and non-inflamed segments of the colon were not attributable to specific taxa. We also observed significant disease-associated reductions of *A. hadrus* and an unclassified species of the Lachnospiraceae family. The study extends the observations of earlier reports^40–44^ not only in the relatively large number of paired (inflamed vs non-inflamed) biopsies, but because of its inclusion of a wider array of molecular data including microbiota, host transcriptome, epigenome and genotype. It also provides enhanced molecular resolution with bacterial species classification and the usage of error-corrected reads, as opposed to representative sequences of operational taxonomic units^45^. Furthermore, to address the confounding effects of lifestyle variations, we recorded the potential impact of habitual diet and other potential modifiers. Curiously, habitual diet appeared to have minimal relationship with observed differences in microbiota composition across the study groups, possibly due to its lesser effect on mucosa-adherent bacteria compared with fecal microbiota^34^. We acknowledge that our results apply to only colonic microbiota and cannot be extrapolated to the small bowel; UC is a disorder confined to the colon, and in the case of CD, all of the patients had clinically-predominant colonic involvement. The contention that microbiota disturbances are greater in ileal rather than colonic CD was not tested in the present study.

The colonic microbiota of patients with IBD exhibited extensive heterogeneity and overlap with that of normal subjects, making it unlikely that specific compositional patterns or signatures alone would have diagnostic fidelity. It should be noted that studies outlining the most pronounced microbiota differences between CD and controls have been sampled either directly from the inflamed ileum^38,39^, which harbors a different ecosystem, or, to an even higher degree, from stool of patients with inflamed ileum^36,37^. There remains, of course, the potential to establish microbiota patterns that identify disease subsets of clinical relevance. Heterogeneity of microbial composition in IBD may arise, in part, because of heterogeneity of host genotype and of the microbiota prior to disease onset. Sub-groups of mixed combinations of CD, UC, and controls have previously been reported^37,46,47^. However, the dominance of particular dominant species in some of the microbiota sub-groups is further testimony to disease heterogeneity, and illustrates the potential impact that single species can have on the community-wide microbiota. Host DNA methylation and gene expression both displayed more pronounced gradients of inflammation than what was observed with the microbiota, suggesting that epigenetic factors mediate interactions between colonic microbiota and host gene expression. Microbiota-sensitive epigenetic signatures were recently observed for histone methylation in CD^48^. While the largest difference in methylation patterns was attributed to inflammation, as expected due to disproportionate composition of inflammatory cells^49^, we did observe significant epigenome-wide differences between CD and control groups (visualized by principal component 6 in Fig. 4). Moreover, a large number of immune-related methylation changes were unevenly distributed across four microbiota clusters, again, potentially affecting microbiota disease heterogeneity. Sub-types of IBD have recently been characterized based on host methylation and expression^49^, but this study also links microbiota-derived sub-groups of IBD with host DNA methylation and transcription.

Our combinatory machine-learning analysis also indicated that microbiota composition together with diet and genotype (even if too few samples for GWAS) were better at classifying disease sub-types than microbiota alone, and that combining microbiota with epigenome data boosts the power even further. While our study is based on a single time-point, longitudinal collections of biopsy samples would likely improve the classification power and also allow for predictive modeling.

Our species-level resolution allowed detection of anti-correlated *B. dorei* and *B. vulgatus* (Fig. 1a), which are indicative of species-specific niche colonization of colonic crypts^50^, and/or competition for dietary inulin believed to reduce inflammation^51^. The only classifiable species significantly less common in both forms of IBD was *A. hadrus*, whose butyrate-producing capabilities^52^ may be protective of colonic inflammation^53,54^. *Ruminococcus bromii* and *Eubacterium rectale* stimulate the growth of *A. hadrus* through cross-feeding from resistant starch breakdown products^55^. Interestingly, we observed the same trend with these symbiotic species whose abundance decreased along PC1 away from controls (Fig. 1a; Supplementary Table 2). *Anaerostipes* has also been reported as one of six genera with lower abundance in stool from patients with CD^56^. Contrary to this, conventional mice gavaged with *A. hadrus* produced more butyrate, but when mice were challenged with a colitis stimuli, they presented an aggravated disease phenotype^57^. The relevance of this and our findings are yet to be addressed.

The reason why the inflammation-specific differences observed in this study (community-wide and within patients), but not in other smaller studies^40–44^, did not translate to differential taxa may be due to the ‘oxygen hypothesis’^58^. Here, oxygen introduced into the gastrointestinal tract as a result of inflammation causes microbial changes regardless of whether a particular site is inflamed. Cross-contamination between inflamed/non-inflamed sites (albeit forceps washed between biopsies), and/or microbiota homogenization following pre-endoscopy bowel preparation, may also contribute^40^. Another consideration is the possible impact of the bowel prep prior to colonoscopy, which may alter fecal (not mucosal) microbial composition^59^, but this was uniform for all patients with active and inactive disease. Some of the microbiota sub-groups were dominated by anaerobic and mutually exclusive *Bacteroides* species. These can, in contrast to most *Escherichia*/*Shigella*/*Klebsiella* species, exert either commensal, mutualistic or pathogenic behaviors depending on host-microbe interactions, bio-geographical location and nutritional availability. As a known pathobiont in IBD, *B. vulgatus* (enriched in cluster 3 subjects) activates NF-kB pathways and some strains are important for colonization and persistence in CD^60^. Similarly, entero-toxigenic *B. fragilis* (dominant species in cluster 1) has been shown to promote intestinal inflammation and possibly colon carcinogenesis through activation of NF-kB^61^. This would result in increased pro-inflammatory cytokine levels such as IL-8/CXCL8, which we were able to confirm in vitro for *Bacteroides vulgatus*. Such rapid changes in host DNA methylation has previously been observed in transcriptional response to pathogenic bacteria^62^.

Similarly, Enterobacteriaceae species are commonly reported as enriched in IBD (see for review ref. ^63^), in particular adherent-invasive *E. coli*, for which we also detected increased chemokine secretion (IL-8/CCL20 levels). A recent study showed that antibiotic-resistant Klebsiella species, which has 100% 16S rRNA gene sequence identity to the *Escherichia*/*Shigella*/*Klebsiella* RSV dominant in cluster 2, can from the oral cavity colonize the gut and elicit inflammation in genetically susceptible hosts^64^.

In conclusion, host-microbe interactions in human IBD are far more complex and heterogeneous than those observed in experimental, inbred, rodent models. Disturbances of the microbiota are evident in both forms of IBD, particularly CD, but further disease stratification is likely to require a higher degree of resolution of microbiome analysis coupled with, and matched for, lifestyle factors, heterogeneity of the host genotype and epigenome.

## Methods

### Study population

The study subjects were all undergoing colonoscopy or sigmoidoscopy as part of their ongoing clinically required care, and volunteered to provide additional biopsy material for research at either Cork University Hospital or the Bons Secours Hospital Cork. These were unselected, consecutively assessed patients with Crohn’s disease (CD) and ulcerative colitis (UC), and were referred for colonoscopy on the basis of standard of care, which was either for dysplasia surveillance as the average duration of disease was ~10 years or for assessment of colonic inflammatory disease activity. Following bowel preparation with MoviPrep according to the manufacturer’s instructions, endoscopists were asked to take samples from areas of active (lesions) inflammation and from normal-appearing areas. This was a binary task (active inflammation vs non-active areas) and no attempt was made to assess the degree of inflammation. For those with CD, colonic biopsies were taken from areas of macroscopically active inflammation (lesional) and from non-inflamed areas (non-lesional) (*n* = 50 biopsy pairs). In the case of patients with sub-total ulcerative colitis (UC; procto-sigmoiditis or left-sided colitis) paired biopsies were taken from the distal inflamed and proximal non-inflamed segment; *n* = 80 biopsy pairs). In all cases, the endoscopic macroscopic interpretation of lesional active inflammation was correlated with histology and in no case was there any disagreement. However, an additional 10 subjects (6 from patients with CD and 4 from UC) were initially enrolled but each had one of their colon biopsy samples excluded from analysis due to inadequate or insufficient material or sequencing reads. All patients had well-established diagnoses by conventional criteria^65^. For both disorders this included consistent clinical features and exclusion of other differential diagnoses in addition to the demonstration of chronicity with relapses and remissions over time. For ulcerative colitis, evidence of inflammation was documented in all cases by prior colonoscopy and histology. In the case of Crohn’s disease, colonic involvement was likewise confirmed colonoscopically, and associated small bowel involvement, where relevant, had been previously shown by computed tomography (CT) and/or by magnetic resonance imaging (MRI). Only one individual refused to participate in the study.

The 32 healthy controls consisted primarily of subjects undergoing colonoscopy for cancer screening or in whom no significant colonic or gastrointestinal disorder was found. In particular, conditions such as irritable bowel syndrome were excluded because of reports of their association with abnormalities of the microbiota^66^. As with the patients with CD and UC, paired biopsies from different colonic segments were taken from all but one of the controls. Long-term dietary habits were captured using food frequency questionnaire based upon the SLAN study^35^. The 147 food items were grouped into ordinal data (number of times consumed per day) from 28 larger food categories. The clinical demographic data on the study subjects is shown in Table 1. The study was approved by the Cork hospital ethics committee and written informed consent was provided by all patients.

### Sample processing, sequencing, array, and in vitro experiments

Immediately after obtainment, biopsies were introduced in 5 mL polypropylene tubes (Sarstedt, Nümbrecht, Germany) that were previously filled with 3 mL of RNA-later (Qiagen, Hilden, Germany). Separate disposable forceps were used in all cases. Samples were kept at 4 °C for 24 h and afterwards stored at −80 °C until nucleic acid purification. Biopsies in RNA-later were completely defrosted before performing DNA/RNA purification using the AllPrep DNA/RNA Mini kit (Qiagen). Briefly, biopsies were extracted from the RNA-later and transferred into a tube with 350 µL of RLT buffer containing β-mercaptoethanol (Sigma-Aldrich, St Louis, MO, USA), three 3.5 mm glass beads and 0.25 mL of 0.1 mm glass beads (Biospec, Bartlesville, OK, USA). Disruption and homogenization was carried out in a MagNA Lyser (Roche, Penzberg, Germany) twice for 15 s at 6500 rpm followed by DNA/RNA purification according to the kit manufacturer’s instructions. Purified genomic DNA was finally eluted in 100 µL of EB buffer, while RNA was eluted in 60 µL of RNase-free water. DNA and RNA concentrations were measured using a Nano-Drop 2000 Spectrophotometer (Thermo Scientific, Waltham, MA, USA) and subsequently samples stored at −80 °C.

Human intestinal epithelial cells CaCO2Bee1 were grown in high-glucose Dulbecco’s modified Eagle’s medium (DMEM; Sigma) supplemented with 10% heat-inactivated fetal bovine serum (FBS; Sigma), 1% penicillin/streptomycin (Pen/Strep; Sigma), and 0.01% transferrin (Sigma). After trypsinisation, cells were seeded into 24-well plates and incubated until ~90% confluent followed by an overnight in serum free medium. Cells were then infected with *B. vulgatus* ATCC8482, *B. fragilis* ATCC25285, *E. coli* HM605-AIEC strain, *S. typhimurium* (bacteria positive control), and a non-inflammatory bacteria strain *Lactobacillus rhamnosus* GG (LGG; APC Culture Collection) at 10:1 multiplicity of infection (MOI) and cultured for 3 h, followed by three times washing with Pen/Strep solution followed by a further 13-h culture in DMEM supplemented with 10% FBS and 1% Pen/Strep. After incubation, supernatants were collected and levels of IL-8/CXCL8 and CCL20/MIP3A levels were measured using an IL-8/CXCL8 ELISA Duo-Set and CCL20/MIP-3 alpha ELISA Duo-Set from R&D Systems as per manufacturer’s instructions.

Library preparation for 16S rRNA gene amplicon sequencing was performed following the Illumina (San Diego, CA, USA) recommendations with some modifications. Briefly, aliquots of 200 ng of extracted DNA was subjected to PCR amplification of the V3-V4 hypervariable region of the 16S rRNA gene in a total volume of 30 µL. The primers (forward TCGTCGGCAGCGTCAGATGTGTATAAGAGACAGCCTACGGGNGGCWGCAG; reverse GTCTCGTGGGCTCGGAGATGTGTATAAGAGACAGGACTACHVGGGTATCTAATCC) had Illumina adapters with containing overhang nucleotide sequences added to the gene-specific sequences^67^ and were used at a concentration of 0.2 µM. PCR amplification with the Phusion High-Fidelity DNA polymerase (Thermo Scientific) was performed on a 2720 Thermal Cycler (Applied Biosystems, Foster City, CA, USA) under the following conditions: 98 °C for 30 s, followed by 30 cycles of 98 °C for 10 s, 55 °C for 15 s, 72 °C for 20 s and a final cycle of 72 °C for 5 min. The presence of the amplified 16S rRNA gene band was verified in agarose gels. Post-PCR products were purified using Agencourt AMPure XP magnetic beads (Beckman-Coulter, Brea, CA, USA) and eluted in 50 µL of EB Buffer (Qiagen). After purification, 5 µL of DNA was amplified in a second PCR employing Nextera XT Index primer (Illumina). This PCR was run at 98 °C for 30 s, followed by 8 cycles of 98 °C for 10 s, 55 °C for 15 s, 72 °C for 20 s and a final cycle of 72 °C for 5 min. A second purification step with Agencourt AMPure XP magnetic beads was carried out after the Nextera PCR. The 16S V3-V4 rRNA gene amplicons containing the Nextera indexes were finally eluted in 25 µL of EB Buffer, and DNA concentrations were measured using Quant-iT Picogreen dsDNA assay kit (Thermo Scientific). Total amplicon yields ranged from 50 ng to 1 µg or ~100 billion to ~2 trillion molecules (400 bp at 660 g/mole/bp). Pooled libraries were created by adding 50 ng of each sample. Finally, diluted (30 nM) samples of the libraries were sent for sequencing at Eurofins Genomics on an Illumina MiSeq for 2 × 300 bp reads.

Prior to genotyping, DNA aliquots were sent on dry ice to Macrogen (Seoul, South Korea) to be assayed on an Infinium ImmunoArray-24 v2 BeadChip. Intact genomic DNA was diluted to 50 ng/µL based on concentrations measured using Quant-iT Picogreen. All samples were hybridized on the microarray according to the manufacturer’s instructions. Briefly, whole genome amplification (×1000) was carried out in 200 ng of DNA. Subsequently, the products were fragmented, precipitated and re-suspended in a formamide-containing hybridization buffer. Next, samples were denatured at 95 °C for 20 min, and then placed in humidified containers for a minimum of 16 h at 48 °C allowing SNP loci to hybridize to the 50mer capture probes. Following hybridization, the BeadChip/Te-Flow chamber assembly was placed on the temperature-controlled Tecan Flowthrough Rack, where subsequent washing, extension, and staining steps were performed. After staining, the slides were washed with a low salt wash buffer, immediately coated, and then imaged on the Illumina iScan Reader. Image intensities were extracted using Illumina’s GenomeStudio software.

Prior to epigenetics analysis, DNA aliquots were sent on dry ice to Hologic (Manchester, UK) where they were assayed on Illumina Infinium HumanMethylation450 BeadChips. Briefly, bisulfite modification was performed using 96-well EZ DNA methylation kit (Zymo Research) with 650 ng of DNA sample. Methylation levels were detected using the Infinium 450 K and the intensity images captured by GenomeStudio (2011.1) Methylation module (1.9.0) software.

Prior to RNA-Seq experiments, RNA samples were processed with DNase to remove any DNA traces employing Turbo DNA-free kit (Ambion) following manufacturer’s instructions. To determine RNA integrity, 1 µL of each RNA sample obtained after DNase treatment was loaded on RNA 6000 Nano Chip (Agilent Technologies) according to the manufacturer’s protocol. RNA integrity number (RIN), rRNA ratio, and concentration were determined by microfluidic capillary electrophoresis in a 2100 Bioanalyzer system (Agilent Technologies). Quality was considered acceptable if RIN was ≥6 and rRNA ratio ≥1.5. Aliquots of RNA samples were sent on dry ice to Macrogen (South Korea) where RNA-Seq was carried out for host transcriptomics. First, a library was generated from 100 ng of total RNA were used for sequencing libraries using TruSeq Stranded mRNA Sample Prep Kit (Illumina), which included an initial step to select and purify polyadenylated RNA (primarily mRNA) employing oligo-dT-conjugated magnetic beads Subsequent experimental procedures were similar for both approaches. Briefly, RNA was purified, fragmented and primed for cDNA synthesis with random hexamers. Actinomycin D was added to suppress DNA-dependent synthesis of the second strand. This was followed by second strand cDNA synthesis using DNA polymerase I, RNase H and dUTP. These cDNA fragments underwent an end repair process, the addition of a single ‘A’ base, and ligation of the adapters. The products were subsequently purified and enriched with PCR to create the final cDNA library. Libraries were analyzed for size distribution using Bioanalyzer (Agilent Technologies), quantified by qPCR according to the qPCR Quantification Protocol Guide (Illumina) and qualified using the TapeStation D1000 ScreenTape (Agilent Technologies). Indexed libraries normalized to 2 nmol/L were sequenced in an Illumina Hiseq 4000 for 2 × 150 bp reads.

### Bioinformatic and statistical analysis

For all sequence data, the quality of the raw reads was visualized with *FastQC* v0.11.3 followed by read trimming and filtering with *Trimmomatic* v0.33^68^ to ensure at least an average quality of 25 and a minimum length of 50 bases after adapter removal, with the reads for 16S rRNA being further filtered following merging of forward and reverse reads. The reads were then imported into R v3.3.0 for analysis with the *DADA2* package (v1.03)^69^. Quality filtering and trimming was performed on both forward and reverse reads with reads only retained when both were of sufficient quality. DADA2 error correction was carried out on each forward and reverse reads separately and subsequently merged, before bimeras were removed from the retained high quality merged reads of at least 340 nucleotides. The resulting unique (as opposed to reads clustered into operational taxonomic units) and error-corrected ribosomal sequence variants (RSVs) were exported and further chimera filtered using an reference based chimera filtering implemented in *USEARCH* v8.1.1861^70^ with the *Chimera-Slayer* gold database v20110519^71^. The non-chimeric RSVs were subsequently classified with the *RDP-Classifier*^72^ in *mothur* v1.34.4^73^ against v11.4 of the RDP database^74^, and *SPINGO*^75^ to species level wherever possible. Only RSVs with a domain classification of Bacteria or Archaea were kept for further analysis. All statistical analysis was carried out in R v3.3.0. Alpha diversity and Bray–Curtis distances were generated using *PhyloSeq* v1.16.2^76^, where principle coordinates analysis was generated using the R package Ape v3.5. Differential taxonomic abundance analysis was carried out with *metagenomeSeq* v1.14.2 with a zero inflated log-normal mixture model. Inferred functional capacity was carried out using *Piphillin*^3^ Hierarchical clustering was performed on the Bray–Curtis distances using the *made4* package v1.46.0^77^, and the number of clusters was decided using the ‘*cuttreeHybrid’* function in *dynamicTreeCut* v1.63^78^. This method was demonstrated to both outperform static height cut-offs for hierarchical clustering, and k-means methods such as Partitioning Around Medoids (PAM) which can favor assigning memberships to large clusters over smaller. Co-variation between ordinal dietary data and RSV abundance profiles was assessed by Procrustes analysis as implemented in *Vegan* v2.3, and Healthy Food Diversity^79^ was compared with alpha diversity using Spearman correlation. Spearman correlations were used for associating metadata and alpha diversity. For healthy controls, the sample with the largest number of reads was selected. For statistical testing of the complete cohort, one sample from each IBD patient was chosen at random, while healthy samples were treated as above. Differences between factors and clusters were examined using a Fishers test for binary/nominal data (metadata) and using a Dunns test for continuous data (dietary information). Spearman correlations between the PC axes with the metadata were also carried out with *adonis*. Reported *p*-values throughout were subjected to Benjamini–Hochberg correction for multiple testing.

The Poly-A captured RNA-Seq reads were aligned to the human genome (GRCh38) using *HiSat* v2-2.0.4^80^. Mapped reads were counted using *featureCounts* v1.5.0^81^. Transcript counts were tested for differential expression using the R package *DESeq2* v.1.10.1^82^ using a paired-sample model for patient replicates. Reported *p*-values throughout were subjected to Benjamini–Hochberg correction for multiple testing. The PCA was created from transformed counts using DESeq2’s *‘regularized log’* transformation. Principle Component Analysis was done with the *prcomp* function on variance stabilized transformed counts as produced by *DESeq2* v1.12.4 and visualized using *ggplot* 2.2.1.

Genotypes were available for 139,193 SNPs (142,662 before QC) on all individuals for use in a host genome association analyses; all individuals had a genotype call rate of >95%. SNPs that deviated from Hardy–Weinberg equilibrium (HWE; *p* < 10^−5^) or with a minor allele frequency (MAF) < 5% were not considered further. Following quality control checks, genotypes on 139,193 SNPs remained. Principal Component Analyses of all SNP genotypes revealed no obvious population stratification when age and gender were accounted for (Supplementary Fig. 3b, c); the lack of any population stratification was substantiated by the lack of an association (*p* > 0.05) between the first three principal components and disease status.

For the epigenome-wide association analyses (EWAS), preprocessing and quality control was implemented using R libraries *minfi* and *minifiData*. Beta values were extracted and filtered, using *BMIQ*^83^ for normalization between probe types with R libraries *methylumi* and *wateRmelon*. Probes were removed if the probe sequence mapped to multiple positions in the genome or mapped to sex chromosomes. Probes with missing data were also excluded. Quantile normalization was used to normalize the methylation beta values (to N(0,1)) at each CpG site across individuals. Principal component analysis was conducted on the normalized DNA methylation data to identify variables to be included as covariates and potential batch effects in the DNA methylation dataset. Each possible covariate considered was correlated with the first methylation PC, which explains 18.4% of the variance. A linear mixed regression model was used (library *lme4*) to test for association between clusters and DNA methylation levels at each CpG site, and also inflammation and DNA methylation levels at each CpG site, adjusting for covariates including condition, status, gender, smoking, age, methylation chip, sample position on methylation chip, hospital, and biopsy location as fixed effects, and patient ID as a random effect. Smoking was defined as the regular consumption of cigarettes, cigar or pipe of any frequency was considered to be active smoking. Total abstinence was required to qualify as a non-smoker. There were only 6 ex-smokers defined as total abstinence for up to 1 year. Reported *p*-values had been corrected for multiple testing using false discovery rate (FDR—specifically the Benjamini–Hochberg procedure), adjusting for tests on all 466,209 sites available, for inflamed/non-inflamed and for each cluster (clusters 1–4) individually. The epigenetic association analysis was repeated at each set of significant epigenetic signals reported for microbiome clusters 1, 2, 3, and 4, incorporating the first 10 PCs as covariates in a linear model of epigenetic association, to account for cell heterogeneity. CpG methylation positions were annotated using the Infinium HumanMethylation450 BeadChip Manifest file.

For the Machine Learning approach, *XGBoost*^36^ was used to build, tune and validate classification models for all possible combinations of microbiota (3222 features), host transcriptome (60,675 transcripts), genotype (264 published IBD loci), and diet data (28 food categories), resulting in 64,189 attributes when all were combined. Leave-one-subject-out cross-validation performance assessment was used whereby samples from all subject, but one, were used for training, whereas the samples of the remaining subject were used for testing. At no point was a model used to classify a sample, where another sample from the same subject has been used to build the model. To eliminate within-subject bias (from the paired nature of samples) in every leave-one-subject-out iteration, each sample from the tested subject was separately used. Only one sample per subject was randomly chosen to represent the training data, while ensuring even representation of inflammation status. The main Xgboost parameters tuned for each model were the percentage of attributes taken for growing each tree, the percentage of data taken for growing each tree, the maximum depth of each tree, the learning rate and the number of trees. Receiver operating characteristic (ROC) curves was assessed against null using *roc.test* from the pROC package^84^.

### Reporting summary

Further information on research design is available in the Nature Research Reporting Summary linked to this article.

## Supplementary information

Supplementary Information

Description of Additional Supplementary Files

Supplementary Data 1

Supplementary Data 2

Supplementary Data 3

Supplementary Data 4

Supplementary Data 5

Supplementary Data 6

Supplementary Data 7

Supplementary Data 8

Supplementary Data 9

Supplementary Data 10

Supplementary Data 11

Supplementary Data 12

Supplementary Data 13

Supplementary Data 14

Reporting Summary

## Data Availability

Sequence and array data are available at NCBI BioProject PRJNA398187 and NCBI GEO GSE103027 and GSE105120. The corresponding metadata is available in Supplementary Table 1, including Montreal classification^85^. More detailed descriptive histology will be available upon request in accordance with ethical guidelines.

## Code availability

The syntax used to carry out the analysis is available at https://github.com/ClaessonLabUCC/Colonic-microbiota-is-associated-with-inflammation-and-host-epigenomic-alterations-in-ibd.
