# Supplementary material for: Colonic microbiota is associated with inflammation and host epigenomic alterations in inflammatory bowel disease
Source: Nat Commun. 2020 Mar 23;11:1512. doi: 10.1038/s41467-020-15342-5 (PMC7089947; doi:10.1038/s41467-020-15342-5)
Supplement: Supplementary file 1 — Supplementary Information [file 41467_2020_15342_MOESM1_ESM.pdf]

**Dietary analysis**

Dietary data, collected through a semi-quantitative 147-types food frequency questionnaire (FFQ)[1,2], was obtained from most (77%) subjects (Figure 3). A principal component analysis of food types showed greater spread of dietary habits for patients with CD and UC (Suppl. Fig. 7A) compared to healthy controls. However, the considerable overlap between the three cohorts suggests that diet is not driving (nor confounding) the observed differences in microbiota composition. Moreover, procrustes analysis confirmed that the diet and microbiota were not subject to any significant co-variation (p-value=0.12; Suppl. Fig. 7B). Shannon microbiota diversity did not correlate well with Healthy Food Diversity[3] (lowest p-value=0.57; Suppl. Fig. 7C). White processed bread was the only food type that were found significantly different across the diseases, being lower in healthy controls compared to CD (p-value<0.025) and UC (p-value<7.82e-05). This may reflect the common avoidance of fiber-associated brown bread by the patients with active IBD.


1 Claesson, M. J. *et al.* Gut microbiota composition correlates with diet and health in the elderly. *Nature* **488**, 178-184, doi:10.1038/nature11319 (2012).

2 Harrington, J. *et al.* Sociodemographic, health and lifestyle predictors of poor diets. *Public Health Nutr* **14**, 2166-2175, doi:10.1017/S136898001100098X (2011).

3 Drescher, L. S., Thiele, S. & Mensink, G. B. A new index to measure healthy food diversity better reflects a healthy diet than traditional measures. *J Nutr* **137**, 647-651 (2007).

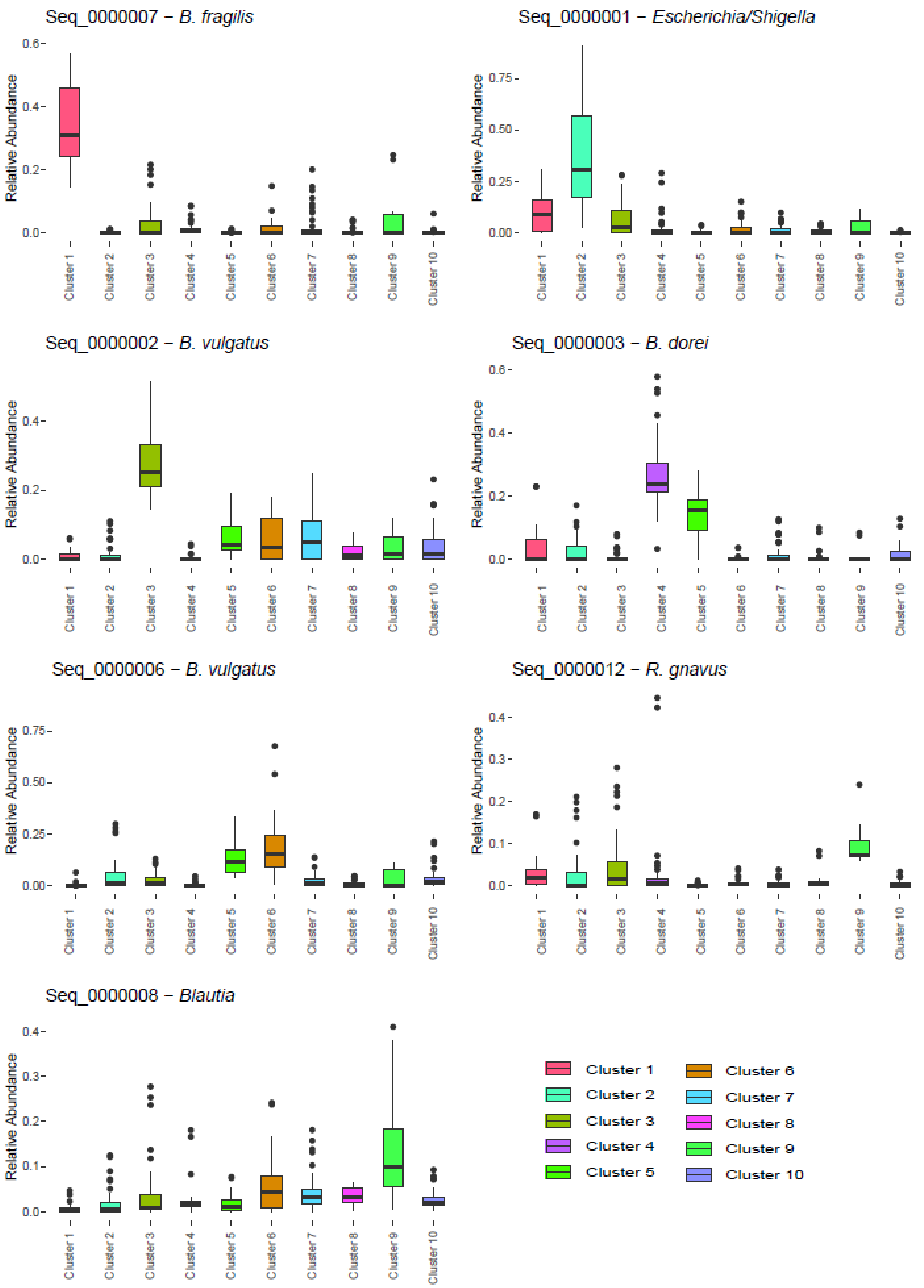


**Supplementary Figure 1.** Box-plots of relative abundances of seven cluster-dominant taxa across the 10 identified clusters (Mann-Whitney test, two-sided; box plot lower and upper sides show 25$^{th}$ and 75$^{th}$ percentiles, respectively. The whiskers are 1.5 of the interquartile range)

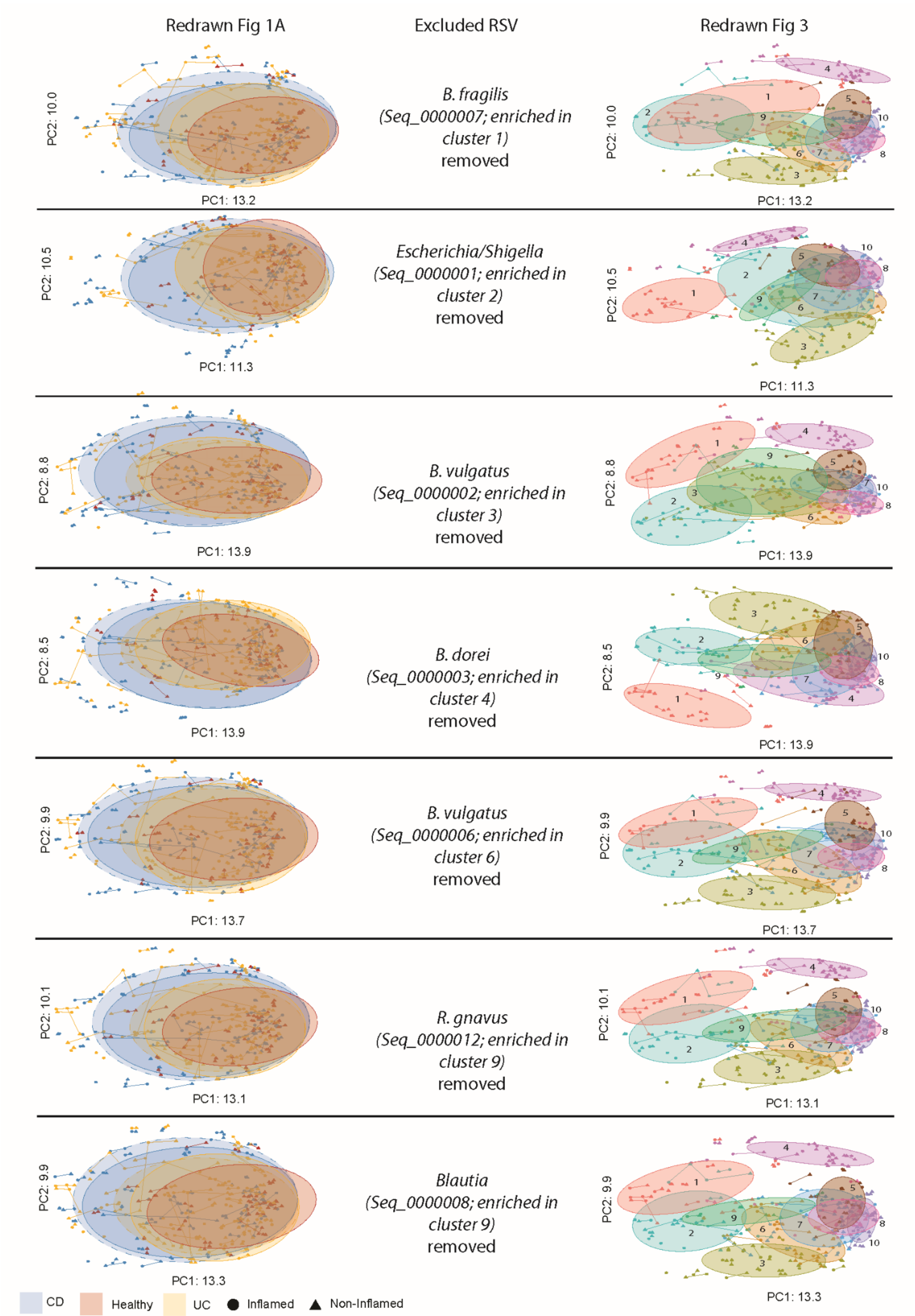


**Supplementary Figure 2.** Investigation of the effect of particular taxa on the overall microbiota composition. Re-drawn PCoAs from Figures 1 and 3 after the seven RSVs were removed one-by-one.

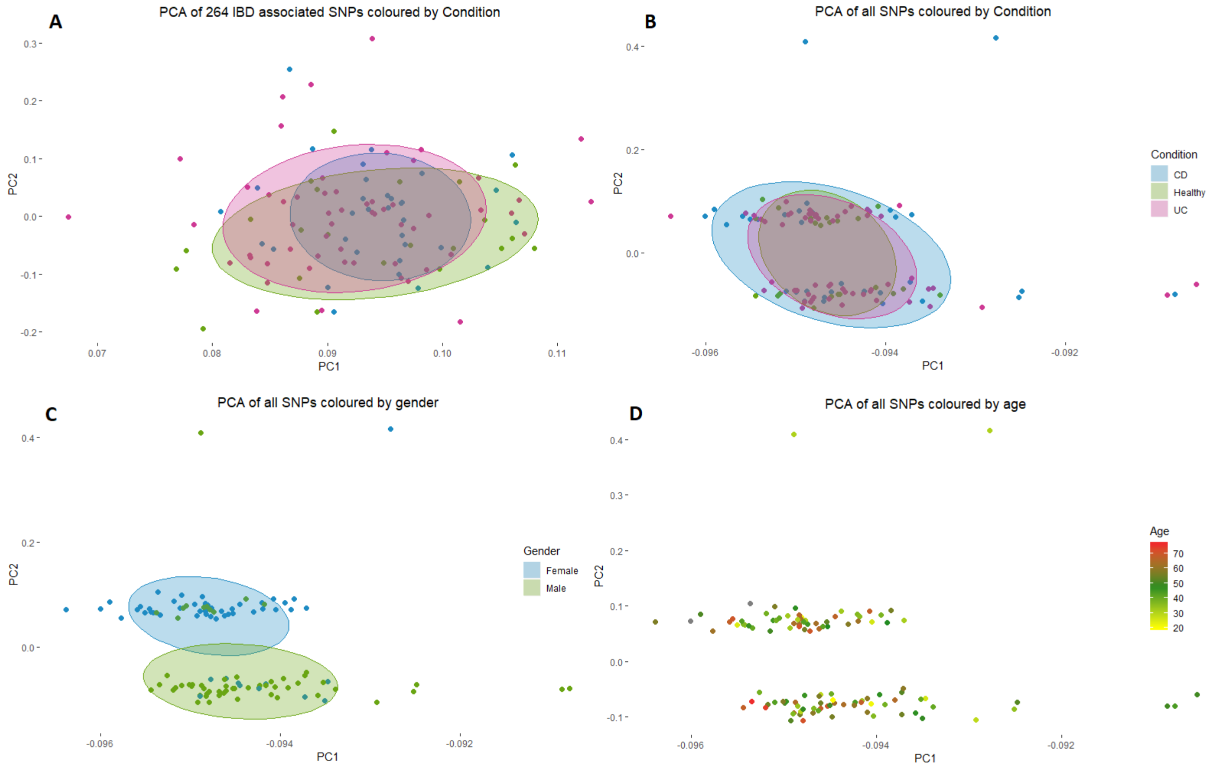


**Supplementary Figure 3.** Principal Component Analysis on nominal categorical data of all known 264 IBD loci for disease (A), and all 139,193 SNPs in the ImmunoArray-24 v2 BeadChip for disease (B), gender (C) and age (D).

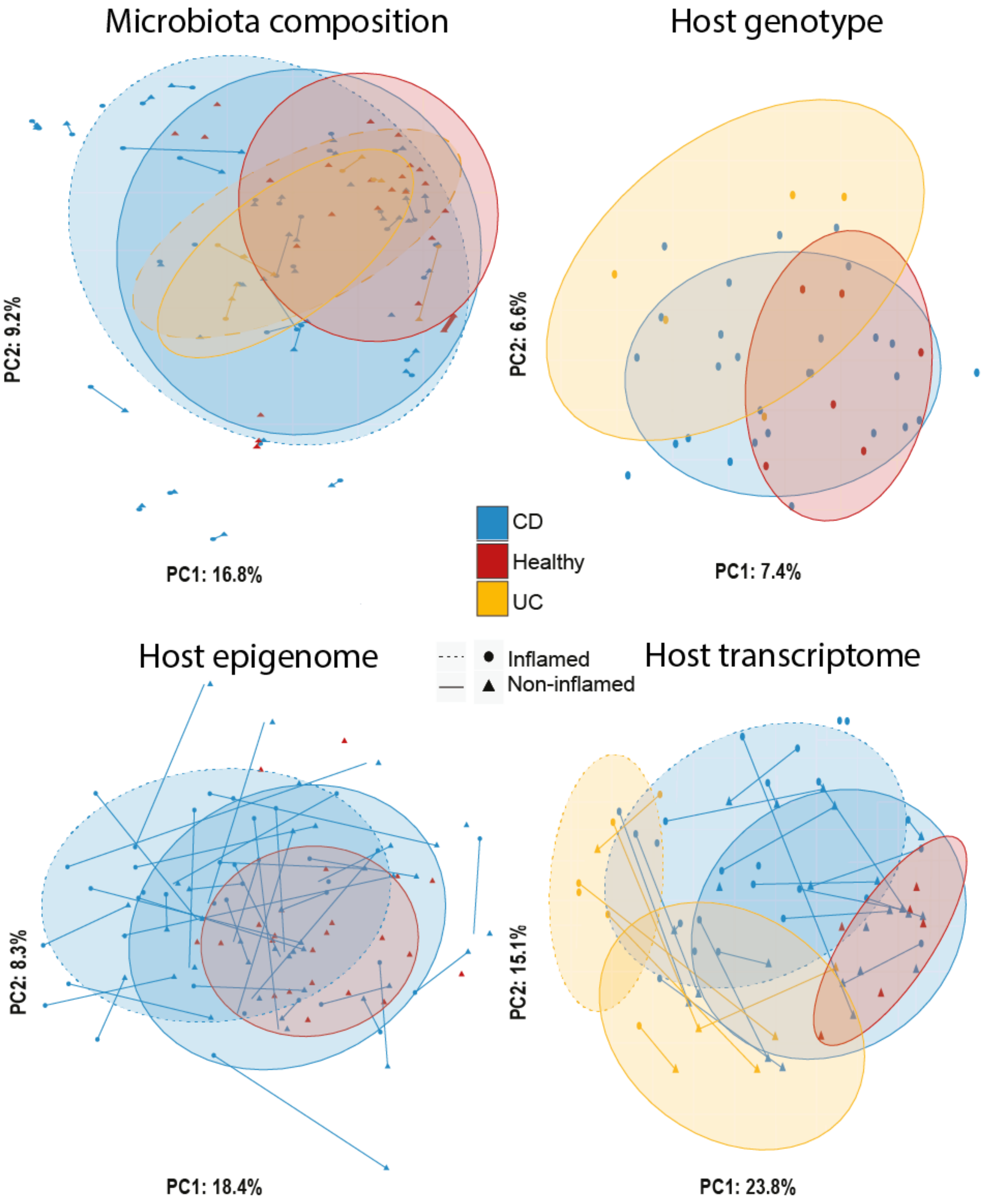


**Supplementary Figure 4.** Ordination plots of 'omics' data from the same subset of samples.

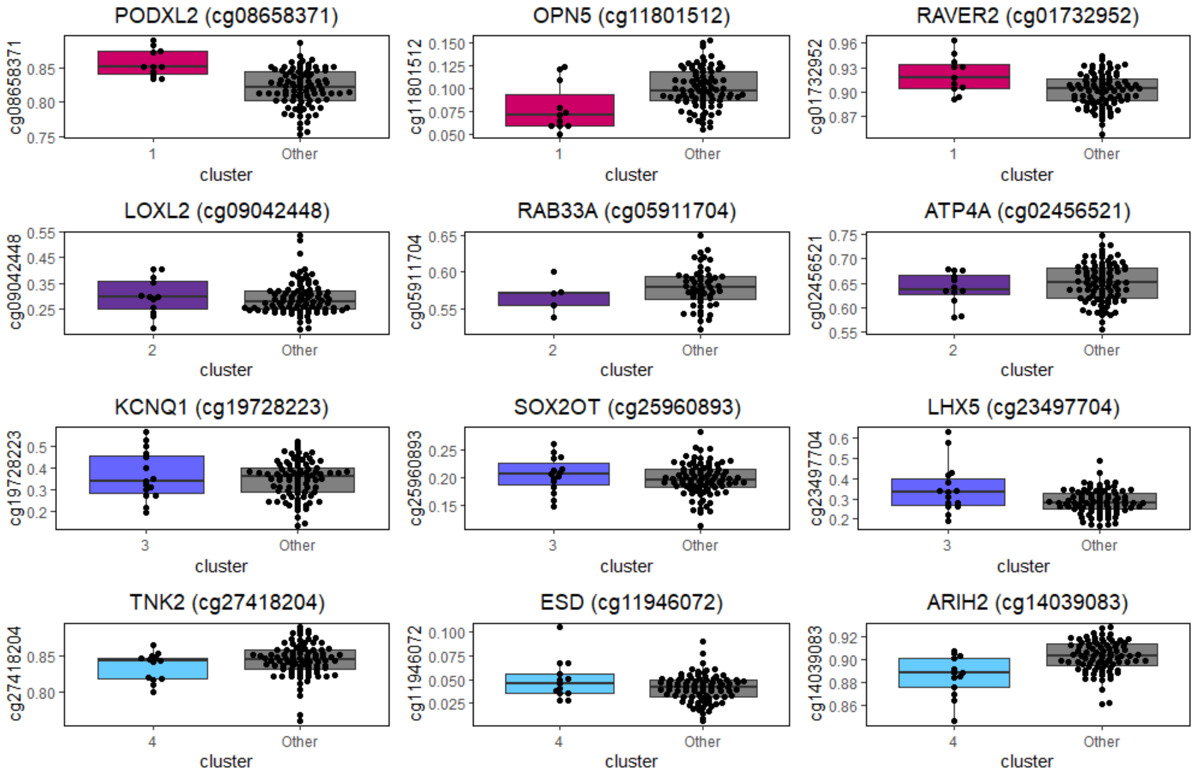


**Supplementary Figure 5.** Significant differential host DNA methylation [beta-values; significance determined using mixed linear models from lme4 library, adjusted with FDR] for 12 of the top (gene associated) CpG sites, in microbiota clusters 1-4. DNA methylation levels at RAB33A on chromosome X are shown for a subset of 58 female subjects (box plot lower and upper sides show 25$^{th}$ and 75$^{th}$ percentiles, respectively. The whiskers are 1.5 of the interquartile range).

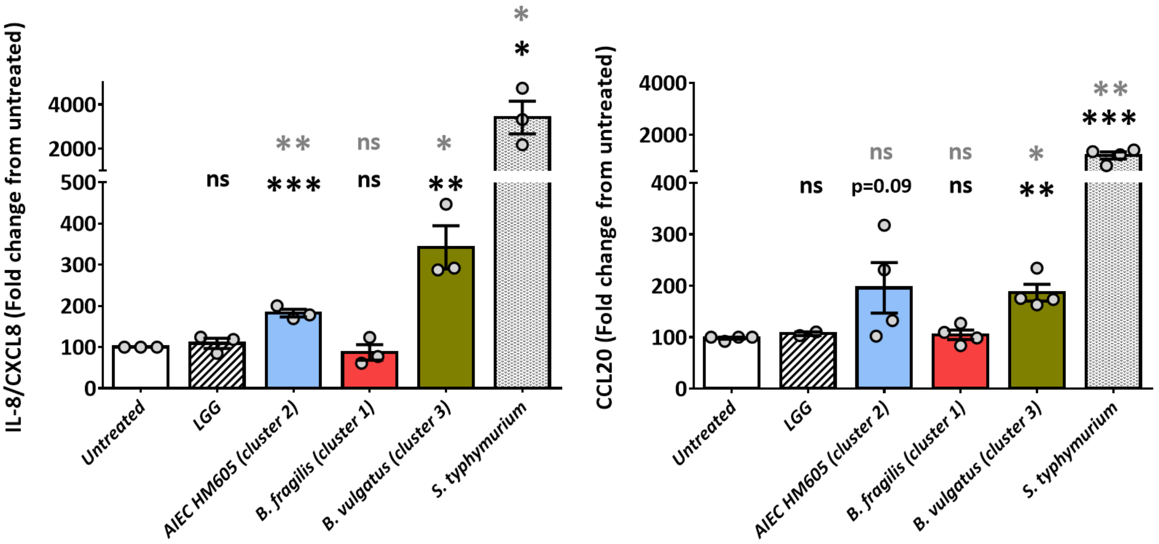


**Supplementary Figure 6. Bacterial type strains representative of the identified microbiota clusters induce a pro-inflammatory response in human intestinal epithelial cells.** CaCO2BBe1 cells were co-cultured with *B. fragilis* ATCC25285 (cluster 1), *E. coli* AIEC strain HM605 (cluster 2), *B. vulgatus* ATCC8482 (cluster 3), an intestinal pathogen *S. typhimurium* ATCC, (bacteria positive control), and the non-inflammatory bacteria strain *Lactobacillus rhamnosus* GG (LGG). Untreated cells were cultured in cell medium alone. Supernatants were collected after 16hrs and analysed for IL-8/CXCL8 and CCL20/MIP3A secretion using ELISA. Data is presented as fold change against untreated cells. Each dot represents one biological experiment made in duplicates or triplicates. Two-sided Student's t test analysis of variance was used to calculate the statistical significance against the untreated cells (black font) and against the LGG strain (grey font). * P < 0.05; ** P <0.01; ***P ≤ 0.001; ns – not significant. Values are expressed as Mean ± SEM, of n=2-4 biological independent experiments per treatment. IL-8: Untreated vs AIEC HM605, p=0.009; Untreated vs *B. vulgatus*, p=0.099; Untreated vs *S. typhymurium*, p=0.0110; LGG vs AIEC HM605, p=0.0089; LGG vs *B. vulgatus*, p=0.0124; LGG vs *S. typhymurium*, p=0.0111. CCL20: Untreated vs *B. vulgatus*, p=0.0016; Untreated vs S. *typhymurium*, p=0.0002; LGG vs *B. vulgatus*, p=0.0306; LGG vs S. *typhymurium*, p=0.0059

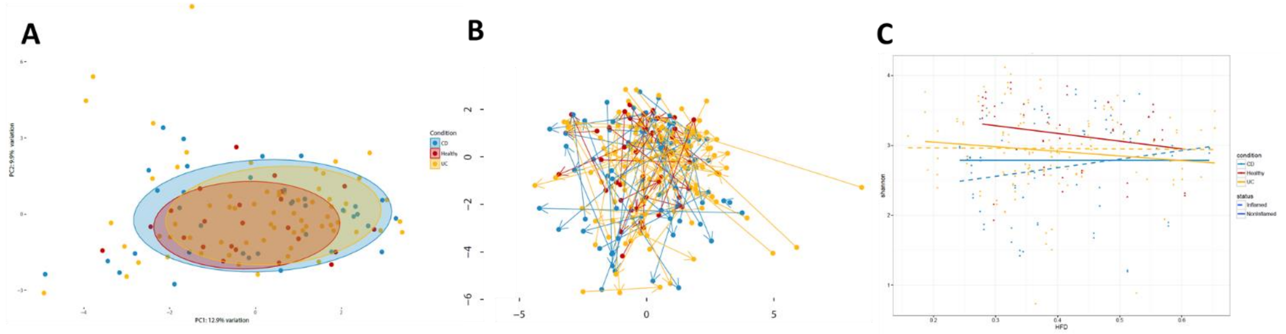


**Supplementary Figure 7.** Dietary analysis with and without microbiota. (A) Principal component analysis of long-term dietary patterns based on ordinal data of 28 major food categories. (B) Procrustes analysis minimizing subject-specific differences between the microbiota PCoA (Figure 1A) and diet PCA (Suppl. Figure 1). The arrow side of each vector represents the microbiota whereas the other side represents the diet for each subject. (C) Scatter plot of Shannon microbiota diversity and Healthy Food Diversity for the three cohorts.

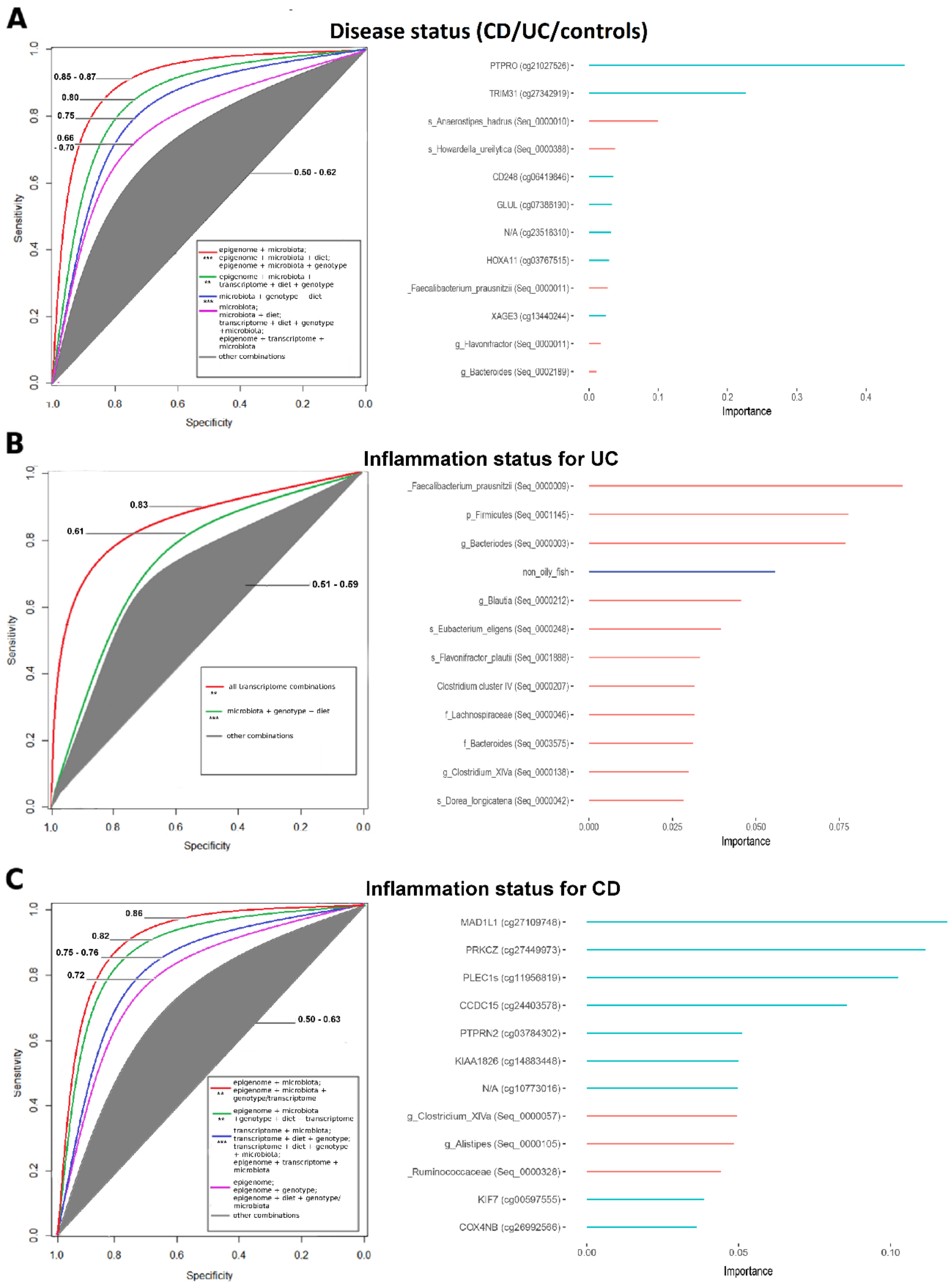


**Supplementary Figure 8.** Left-most column shows Receiver Operator Curves (ROC) classification models of different combinations of cohorts and available data (microbiota composition, host transcriptome, genotype and diet). Healthy controls were excluded when classifying inflammation status. The grey areas represent ROC for models with poor (≤0.6) Area Under Curve (AUC) values to ease visualization. When different combinations have the same AUC, they are represented by the same line. Right-most column shows the top 12 highest weighted features (importance, average gain of each feature across all decision trees within a model) unique to each model built using microbiota (red), diet (green) and genotype (blue) data. First letters indicate (p)hylum, (o)rder, (f)amily, (g)enus, (s)pecies. Epigenome data was not available for UC samples (*roc.test* from library pROC, one-sided; P-values: *<0.0.5; **<0.01; ***<0.001; P-values in Suppl. Table 13).
