# Supplementary material for: Colonic microbiota is associated with inflammation and host epigenomic alterations in inflammatory bowel disease
Source: Nat Commun. 2020 Mar 23;11:1512. doi: 10.1038/s41467-020-15342-5 (PMC7089947; doi:10.1038/s41467-020-15342-5)
Supplement: Supplementary file 2 — Description of Additional Supplementary Files [file 41467_2020_15342_MOESM2_ESM.docx]

Description of Additional Supplementary Files

File Name: Supplementary Data 1

Description: Clinical and disease metadata of subjects and samples

File Name: Supplementary Data 2

Description: Bacterial taxa (RSVs) correlated with the principal components

File Name: Supplementary Data 3

Description: Differentially abundant RSVs between groups

File Name: Supplementary Data 4

Description: Differentially abundant KEGG orthologs (KO) - two-sided test from the DESeq2 package, adjusted p-value was calculated using Benjamini-Hochberg method

File Name: Supplementary Data 5

Description: Differentially methylated CpG sites from inflamed vs non inflamed Crohn's Disease biopsies, and corresponding differential expression results

File Name: Supplementary Data 6

Description: Differentially methylated CpG sites (Crohn's Disease only) from clusters 1 to 4 vs all others, and corresponding differential expression results

File Name: Supplementary Data 7

Description: Differentially expressed genes (Crohn's Disease only) for inflamed vs non-inflamed mucosa

File Name: Supplementary Data 8

Description: Differentially expressed genes (Ulcerative Colitis biopsies) in inflamed vs non-inflamed mucosa

File Name: Supplementary Data 9

Description: GO Ontology results for differentially expressed genes associated with Crohn's Disease inflamed mucosa

File Name: Supplementary Data 10

Description: GO Ontology results for differentially expressed genes associated with Ulcerative Colitis inflamed mucosa

File Name: Supplementary Data 11

Description: GO Ontology results for differentially expressed genes associated with Crohn's Disease non-inflamed mucosa

File Name: Supplementary Data 12

Description: GO Ontology results for differentially expressed genes associated with Ulcerative Colitis non-inflamed mucosa

File Name: Supplementary Table 13.xls

Description: Classification models AUC results for different data type of omics combinations File Name: Supplementary Table 14.xls

Description: Supplementary Table 14: Kruskal Wallis test for experimental batch effects
