# Supplementary material for: Colonic microbiota is associated with inflammation and host epigenomic alterations in inflammatory bowel disease
Source: Nat Commun. 2020 Mar 23;11:1512. doi: 10.1038/s41467-020-15342-5 (PMC7089947; doi:10.1038/s41467-020-15342-5)
Supplement: Supplementary file 17 — Reporting Summary [file 41467_2020_15342_MOESM17_ESM.pdf]

nature research

Corresponding author(s): Marcus J Claesson

Last updated by author(s): Feb 17, 2020

Reporting Summary

Nature Research wishes to improve the reproducibility of the work that we publish. This form provides structure for consistency and transparency in reporting. For further information on Nature Research policies, see Authors & Referees and the Editorial Policy Checklist .

Statistics

For all statistical analyses, confirm that the following items are present in the figure legend, table legend, main text, or Methods section.

n/a	Confirmed	
☐	☒	The exact sample size (*n*) for each experimental group/condition, given as a discrete number and unit of measurement
☒	☐	A statement on whether measurements were taken from distinct samples or whether the same sample was measured repeatedly
☐	☒	The statistical test(s) used AND whether they are one- or two-sided<br>*Only common tests should be described solely by name; describe more complex techniques in the Methods section.*
☐	☒	A description of all covariates tested
☐	☒	A description of any assumptions or corrections, such as tests of normality and adjustment for multiple comparisons
☐	☒	A full description of the statistical parameters including central tendency (e.g. means) or other basic estimates (e.g. regression coefficient) AND variation (e.g. standard deviation) or associated estimates of uncertainty (e.g. confidence intervals)
☐	☒	For null hypothesis testing, the test statistic (e.g. *F*, *t*, *r*) with confidence intervals, effect sizes, degrees of freedom and *P* value noted<br>*Give P values as exact values whenever suitable.*
☒	☐	For Bayesian analysis, information on the choice of priors and Markov chain Monte Carlo settings
☐	☒	For hierarchical and complex designs, identification of the appropriate level for tests and full reporting of outcomes
☒	☐	Estimates of effect sizes (e.g. Cohen's *d*, Pearson's *r*), indicating how they were calculated

Our web collection on statistics for biologists contains articles on many of the points above.

Software and code

Policy information about availability of computer code

Data collection	No software was used for data collection. Methods for sequencing are detailed in the methods section of the manuscript.
Data analysis	The specific data analyses used in this study, including software names and versions, are available at https://github.com/ClaessonLabUCC/Colonic-microbiota-is-associated-withinflammation-and-host-epigenomic-alterations-in-ibd

For manuscripts utilizing custom algorithms or software that are central to the research but not yet described in published literature, software must be made available to editors/reviewers. We strongly encourage code deposition in a community repository (e.g. GitHub). See the Nature Research guidelines for submitting code & software for further information.

Data

Policy information about availability of data

All manuscripts must include a data availability statement. This statement should provide the following information, where applicable:

- Accession codes, unique identifiers, or web links for publicly available datasets
- A list of figures that have associated raw data
- A description of any restrictions on data availability

Sequence and array data iare available at NCBI BioProject PRJNA398187 and NCBI GEO GSE103027 and GSE105120. The corresponding meta-data is available in Supplementary Table 1. More detailed descriptive histology will be available upon request in accordance with ethical guidelines. Data analysis details are available at https://github.com/ClaessonLabUCC/Colonic-microbiota-is-associated-with-inflammation-and-host-epigenomic-alterations-in-ibd

Field-specific reporting

Please select the one below that is the best fit for your research. If you are not sure, read the appropriate sections before making your selection.

- [x] Life sciences
- [ ] Behavioural & social sciences
- [ ] Ecological, evolutionary & environmental sciences

For a reference copy of the document with all sections, see nature.com/documents/nr-reporting-summary-flat.pdf

Life sciences study design

All studies must disclose on these points even when the disclosure is negative.

Sample size	No power calculation was carried for this observational study, as the cohort size was ultimately dependent on how many subjects that could be sampled within the clinical collection period. All results are, however, reported with statistical significance
Data exclusions	An additional 10 subjects (6 with CD and 4 with UC) were initially enrolled, but each had one of their colon biopsy samples excluded from analysis due to inadequate or insufficient material or sequencing reads. The exclusion criteria were established pre-study in the approved ethics protocol.
Replication	All findings are reproducible if the same analysis protocol is applied to the same input data, as per URLs above
Randomization	This is an observational study and not a clinical trial, thus randomization is not applicable. However, samples were randomized on sequencing runs and arrays as is good practice
Blinding	his is an observational study and not a clinical trial, thus blinding is not applicable.

Reporting for specific materials, systems and methods

We require information from authors about some types of materials, experimental systems and methods used in many studies. Here, indicate whether each material, system or method listed is relevant to your study. If you are not sure if a list item applies to your research, read the appropriate section before selecting a response.

Materials & experimental systems

n/a	Involved in the study	
x		Antibodies
x		Eukaryotic cell lines
x		Palaeontology
x		Animals and other organisms
	x	Human research participants
x		Clinical data

Methods

n/a	Involved in the study	
x		ChIP-seq
x		Flow cytometry
x		MRI-based neuroimaging

Human research participants

Policy information about studies involving human research participants

Population characteristics	The study subjects were all undergoing colonoscopy or sigmoidoscopy as part of their ongoing clinically-required care, and volunteered to provide additional biopsy material for research at either Cork University Hospital or the Bons Secours Hospital Cork. The 32 healthy controls consisted primarily of subjects undergoing colonoscopy for cancer screening or in whom no significant colonic or gastrointestinal disorder was found. Further population characteristics can be found in supplementary table 1.
Recruitment	Patients in this study were recruited from Cork University Hospital or Bons Secours Hospital in Cork. There were no self-selection and consented patients were recruited if they presented with inflamed mucosa, as stated in the study design.
Ethics oversight	All patients provided written and oral informed consent approved by the Cork Hospital Ethic Committee (CREC), which is now added to Methods.

Note that full information on the approval of the study protocol must also be provided in the manuscript.
